# Intraventricular sizeable colloid cyst with atypical radiological features: A case report and evidence-based review

**DOI:** 10.1016/j.radcr.2023.07.064

**Published:** 2023-08-15

**Authors:** Moustafa A. Mansour, Dyana F. Khalil, Abdou Hamdi, Mahmoud Bayoumi, Mohamed Abdel-Fattah El-Salamoni, Ali Elsoulia, Ahmed A. Lasheen, Abdelrahman E. Kamel, Mohamed Nawara, Ahmad A. Ayad

**Affiliations:** a Department of Neurology and Neurologic Surgery, Faculty of Medicine, Al-Azhar University, Cairo, Egypt; b Department of Neurology and Neurologic Surgery, Mayo Clinic, Rochester, MN, USA; c Division of Neuro-Intensive Care, Dar Al-Fouad Medical Corporation, Cairo, Egypt; d Department of Emergency Medicine and Critical Care, Faculty of Medicine, Al-Azhar University, Cairo, Egypt; e Department of Oncology, Faculty of Medicine, Al-Azhar University, Cairo, Egypt; f Department of Human Physiology and Basic Medical Sciences, Faculty of Medicine, Al-Azhar University, Cairo, Egypt; g Faculty of Medicine, Mansoura University, Mansoura, Egypt; h Faculty of Medicine, Zagazig University, Zagazig, Egypt; i Faculty of Medicine, Minia University, Minia, Egypt

**Keywords:** Colloid cyst, Third ventricle, Hydrocephalus, External ventricular drain, Histopathology, Neuroradiology

## Abstract

Colloid cysts are benign intracranial lesions, typically located in the anterior portion of the third ventricle near the interventricular foramina of Monro. The cysts usually consist of an epithelial lining filled with viscous gelatinous material of various components. Colloid cysts are generally asymptomatic, but once symptomatic, they can present in a variety of ways, including headaches, vomiting, visual and memory problems, and vertigo. Colloid cysts present classically on imaging as a well-delineated hyperattenuating lesion on unenhanced radiological modalities. Herein, we report a case of a patient who presented with hydrocephalus caused by a sizeable colloid cyst which demonstrated atypical imaging findings in the form of hypodensity on CT and hyperintensity on T2WI, making them difficult to identify and easy to miss. Although this atypical imaging appearance is uncommon with yet unknown true incidence, it is prudent to be aware of it because early management of colloid cysts has a favorable outcome, in contrast to untreated cysts that are associated with higher rates of morbidity and mortality. Additionally, we provide a comprehensive, evidence-based review of the medical entity of intracranial colloid cysts with highlights of current postulated pathological theories and management algorithms.

## Introduction

Colloid cysts are benign intraventricular cysts first described by Wallmann in 1858. Formerly, colloid cysts were a part of the WHO classification of CNS neoplasms, incorporated within the “cysts and tumor-like lesions” category. The true incidence of colloid cysts remains unknown and difficult to estimate because many remain asymptomatic till death [1,2]; however, symptomatic colloid cysts are reported in approximately 1-3/106 person-years [3,4]. Due to their critical anatomical location, these colloid cysts can cause CSF flow obstruction, leading to signs and symptoms of increased ICP, including headaches, vomiting, visual failure, ataxia, vertigo, memory deficits, and sudden death [5]. Therefore, prompt recognition and management of these lesions are crucial. Imaging plays a critical role in the diagnostic step of these colloid cysts and helps decide the most appropriate management option. Typical imaging characteristics of these cysts are classic of a hyperattenuating lesion in the roof of the third ventricle, with or without hydrocephalus [6]. However, some lesions might present atypically. In this clinical article, we report a case of a sizeable colloid cyst associated with severe hydrocephalus and atypical imaging features.

## Case presentation

**Fig. 1:**
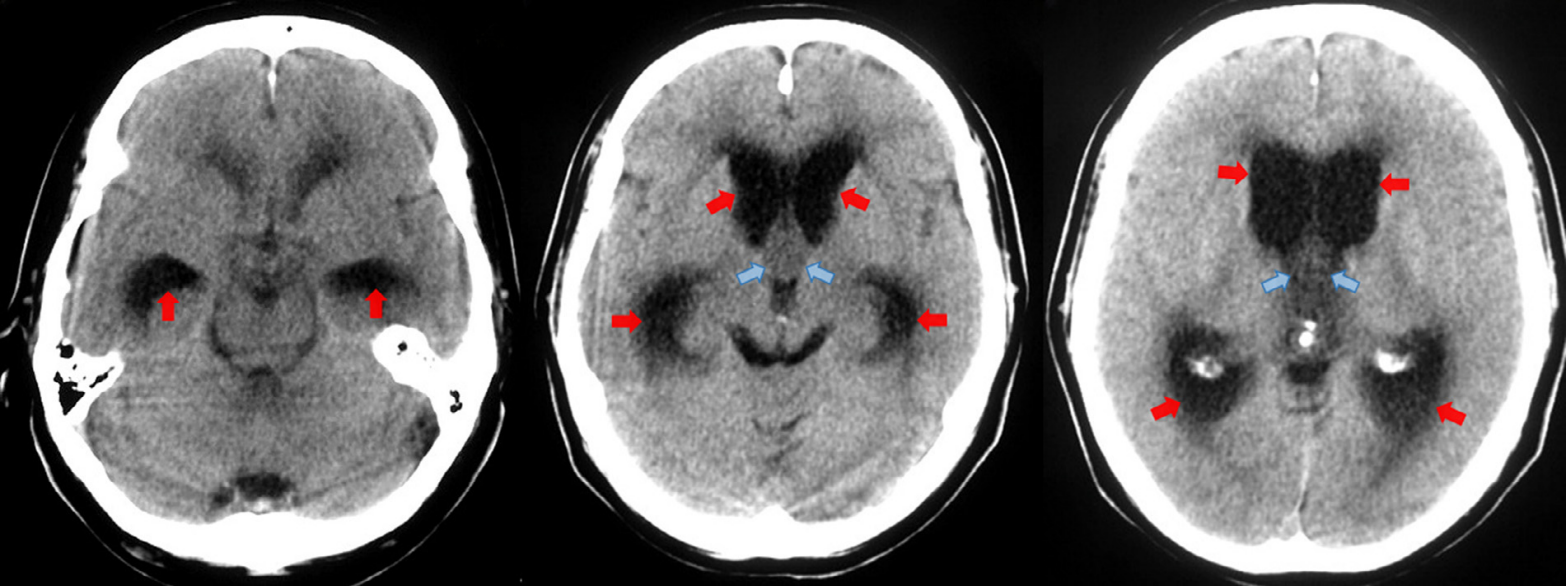
Axial CT images without contrast demonstrate a well-defined homogeneous hypodense nodular lesion (*blue arrows*) located between the foramina of Monro and the anterior portion of the third ventricle, causing symmetrical expansion of the lateral ventricles (*red arrows*).

**Fig. 2:**
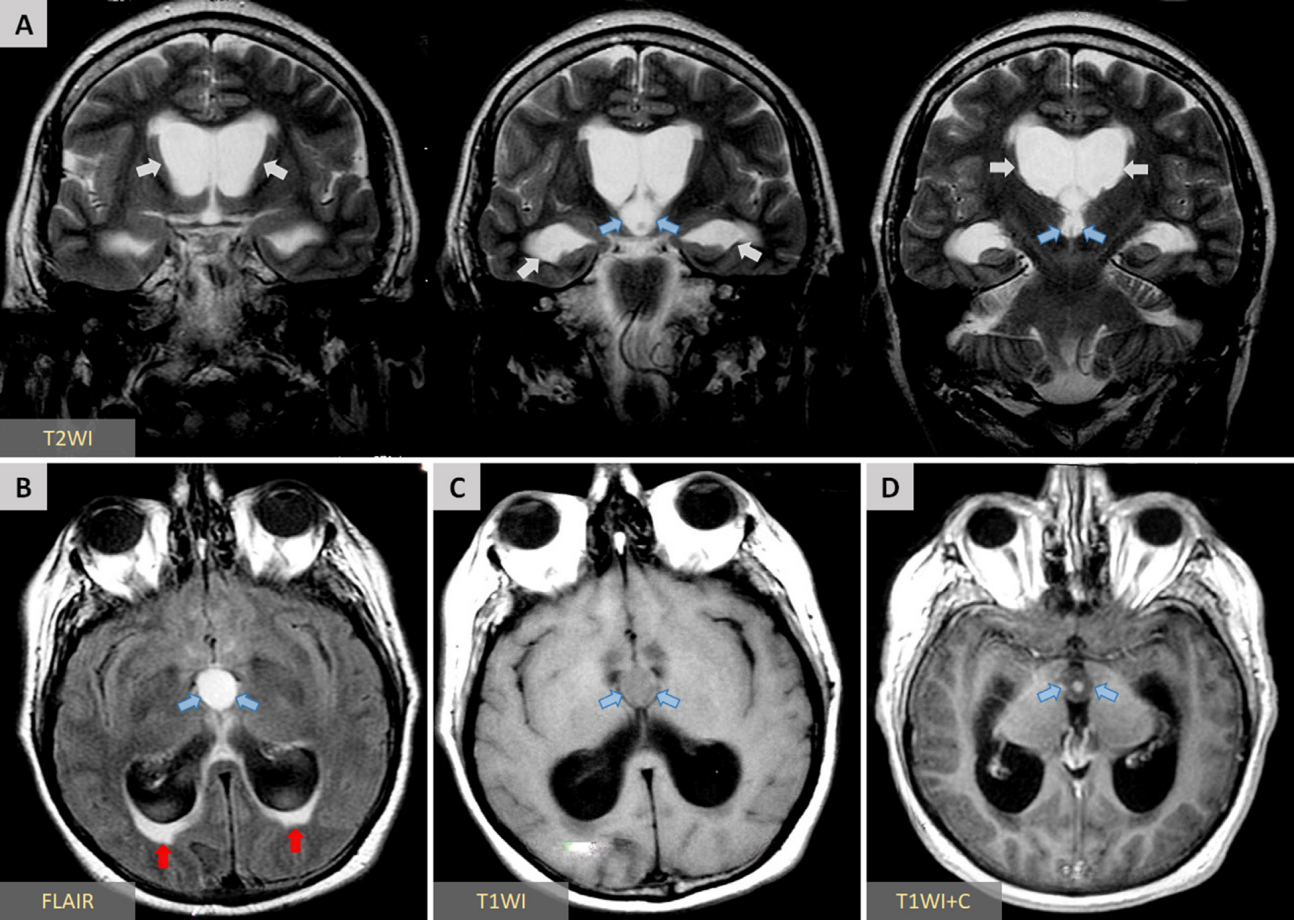
(A) Coronal T2-weighted MR images at different levels better characterize the described lesion as a well-defined fluid-filled cystic mass (*blue arrows*) located in the midline, in the topography of the anterior portion of the third ventricle, obstructing the foramina of Monro with resultant marked hydrocephalus (*white arrows*). (B) Axial FLAIR image demonstrates a still hyperintense signal exhibited by the described lesion (*blue arrows*), even with CSF signal suppression, postulating a higher protein content within the lesion compared to CSF. Some periventricular areas also exhibit hyperintense signals (*red arrows*), consistent with the transependymal flow caused by the existing obstructive hydrocephalus. (C) Axial T1-weighted MR image demonstrates an isointense signal exhibited by the lesion with no signs of enhancement postcontrast administration (*blue arrows*) (D).

**Fig. 3:**
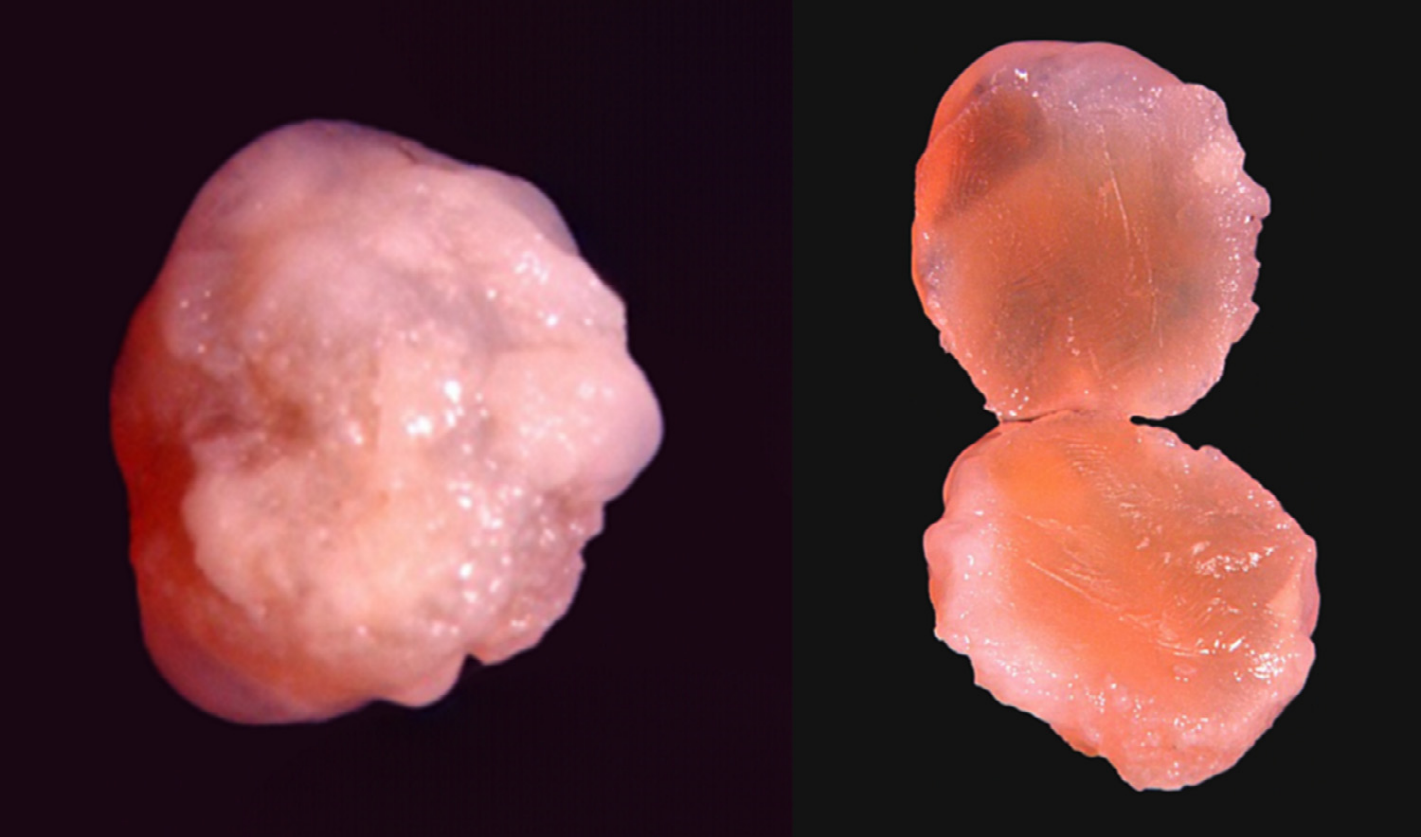
Macroscopic picture of the contents of the lesion approached by endoscopy, after being exposed to controlled freezing and peeling off the covering capsule, revealing mucoid material of different textures and colors.

**Fig. 4:**
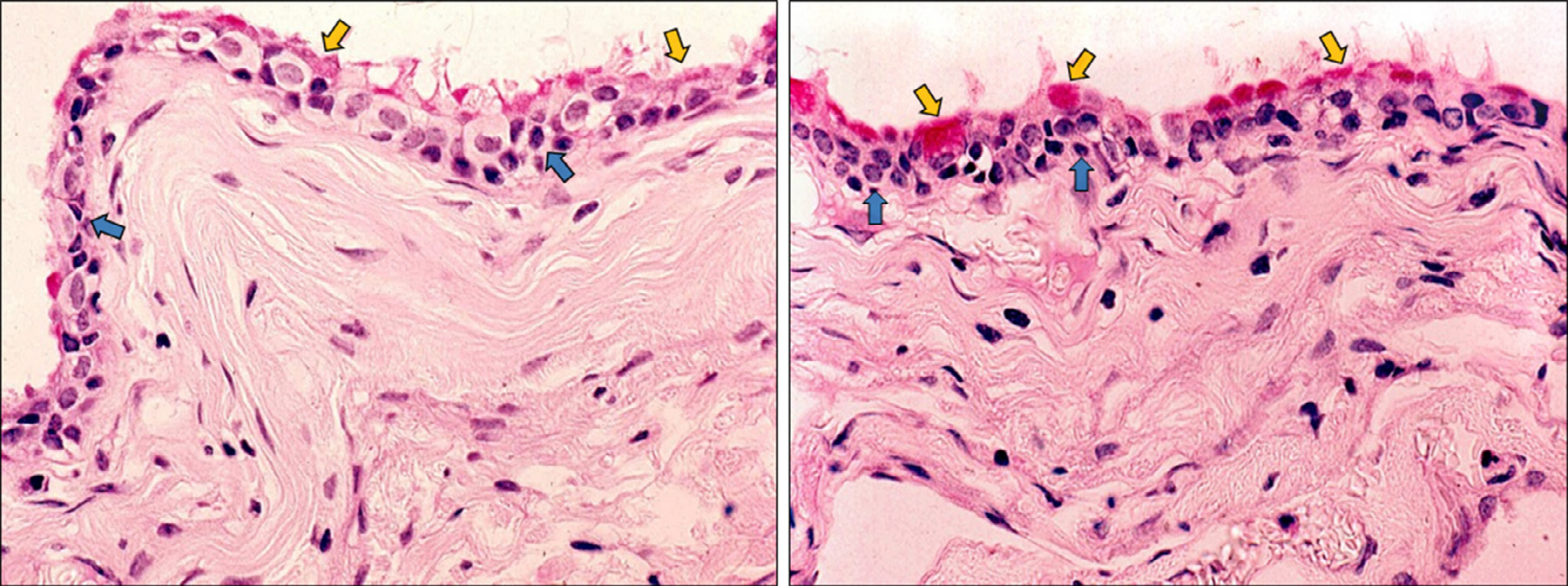
PAS and H&E-stained microscopic images of the peeled capsule reveal a lining pseudostratified ciliated epithelium (*blue arrows*) with a magenta-colored mucoid material (*yellow arrows*).

**Fig. 5:**
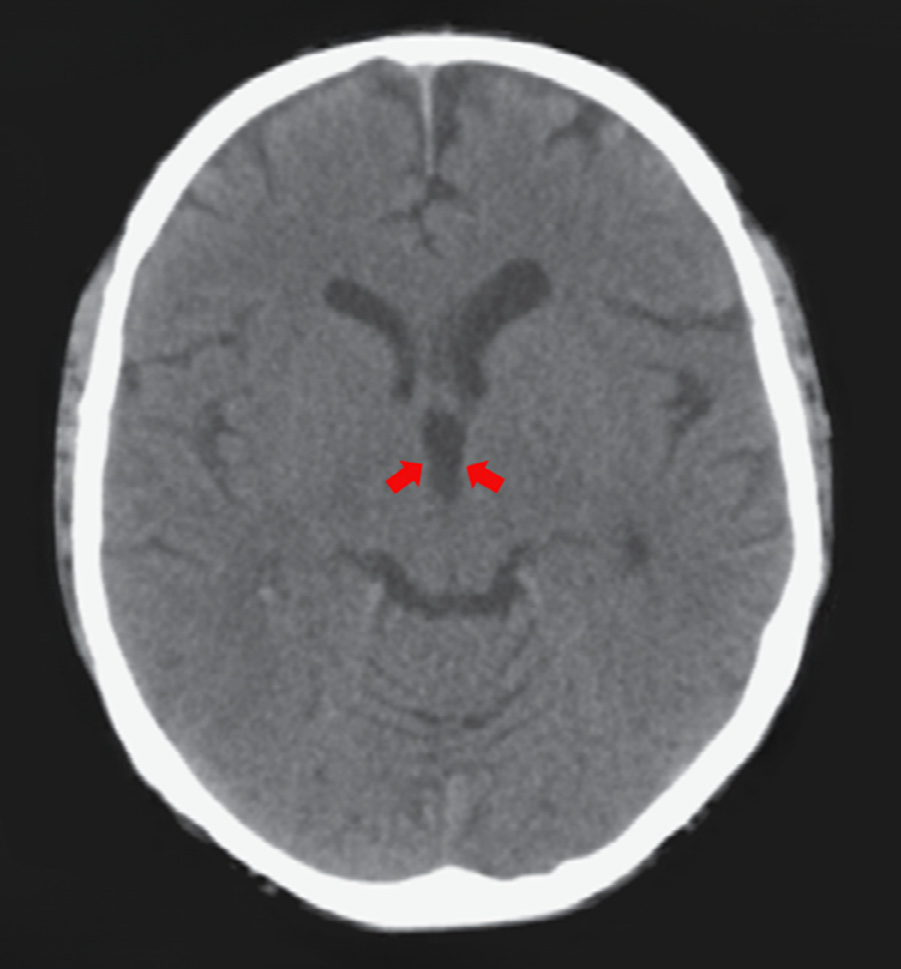
Axial CT image without contrast 7 days postoperatively, showing total cyst excision and reduction of the preoperative hydrocephalus with a completely patent third ventricle (*arrows*).

A right-handed 62-year-old man presented to the emergency department with a 4-week history of progressively worsening global, dull headache. Two days before admission: the patient exhibited intermittent confusion episodes with episodic transient visual obscurations. Direct questioning of the patient and the relatives, who accompanied the patient, revealed that the patient had been experiencing episodic self-limiting dull headaches for the past 3 months but could not attribute these episodes to any specific activity or time of day; therefore, he had not sought medical advice. The patient had an unremarkable medical history, and no family history of particular conditions was evident. The patient was neurologically intact. Fundoscopy revealed bilateral Frisen stage 2 papilledema. A brain CT scan revealed a large well-defined homogeneous nodular lesion between the foramina of Monro and the anterior portion of the third ventricle, causing symmetrical expansion of the lateral ventricles (Fig. 1). The lesion was slightly hypodense compared to the surrounding brain tissue, with no evident calcifications (Fig. 1). T2-weighted MRI studies further characterized the lesion as a fluid-filled midline cystic lesion in the topography of the anterior portion of the third ventricle (Fig. 2A), causing symmetrical obstructive hydrocephalus with a significant transependymal flow (Fig. 2B). The lesion additionally demonstrated an isointense signal on T1WI, with a subtle central hyperintensity (Fig. 2C). Furthermore, the lesion did not enhance postcontrast administration (Fig. 2D). Based on the data obtained thus far, we were able to make a preliminary diagnosis of a sizeable colloid cyst associated with obstructive hydrocephalus. However, the acquired imaging data are atypical of what colloid cysts typically look like. Given the patient's symptoms and the imaging data, we offered the patient endoscope-assisted surgical resection of the lesion. Upon admission, he underwent a right frontal craniotomy with a transcortical transventricular approach to the lesion, which was confirmed as a colloid cyst. The cyst was resected in whole (Fig. 3), and was sent subsequently for histopathological evaluation. An ipsilateral external ventricular drain (EVD) was placed at the end of the operation for an instant alleviation of the high intracranial pressure and was removed 3 days later. Histology revealed fragments of a cyst lined by ciliated pseudostratified epithelium with mild capsular hyalinization and mucoid material of different textures and colors (Fig. 4). A final diagnosis was made of a colloid cyst of the third ventricle with no evidence of malignancy. The patient exhibited unremarkable postoperative recovery. Postoperative CT imaging revealed total cyst resection with progressive reduction of the preoperative hydrocephalus (Fig. 5). The patient is currently well with no new neurologic deficit.

## Case discussion

Thus far, neuropathologists still debate the true origin of colloid cysts. Colloid cysts were thought to originate from the neuroectodermal elements of the paraphysis cerebri [7,8]. However, recent immunohistochemistry studies have challenged the notion of the neuroepithelial origin of colloid cysts by the presence of several different cell types lining the cyst capsule, including ciliated and nonciliated, goblet, basal, and squamous cells, suggesting an endodermal rather than ectodermal origin of these colloid cysts. Therefore, migration of endodermal elements during early brain development with either slow or delayed growth in the roof of the third ventricle has been favored as a critical factor in the etiology of colloid cysts [9].

There are numerous available case reports detailing family histories of colloid cysts [[10], [11], [12]], the issue that made some authors postulate a potential autosomal dominant inheritance pattern of these colloid cysts [13], while others suggested an autosomal recessive pattern of inheritance with variable penetrance [14]. Nonetheless, it remains unclear whether these reported cases will help explain the inheritance of familial colloid cysts; therefore, well-established evidence is still needed.

Colloid cysts can present in a variety of ways, with the most prevalent symptoms being headache (76%), followed by vomiting (24%), visual failure (21%), ataxia (12%), and memory deficit (10%) [5]. Headache is usually a nonspecific symptom, but progressive headache associated with visual obscurations and vomiting merits a scan even if there is no papilledema on examination. Several reports have postulated an association between colloid cysts and sudden death, but the strength of this association is not significant and still controversial. However, the cyst size may be an indicating factor in this regard, especially when greater than 1 cm in diameter [[15], [16], [17]], because these sizeable cysts might have a higher potential for hypothalamic compression and subsequent cardiorespiratory failure.

Imaging studies remain the cornerstone of evaluation for patients with colloid cysts. Classically, colloid cysts demonstrate a well-defined hyperattenuating circular mass on unenhanced CT scans, while on MRI modalities, they usually are hyperintense on T1WI and isointense to hypointense on T2WI. Therefore, colloid cysts with a hypodense signal on CT, an isointensity on T1WI, and hyperintense signals on T2WI and FLAIR sequences, as demonstrated in our case, are not typical for these cysts and are rarely reported, making them easy to miss on routine imaging studies. However, it is prudent to be aware of such atypical findings, especially with the currently available data that suggest that colloid cysts with hyperintensity on T2WI and FLAIR are associated with relatively larger sizes and younger patient age, and hence risk factors for obstructive hydrocephalus [[18], [19], [20]].

Available data suggest serial observations for asymptomatic colloid cysts [21]. However, symptomatic cysts were found to have a 32% incidence of life-threatening progression, requiring prompt management [22]. Colloid cyst risk score (CCRS) was proposed by Beaumont et al*.* [23] in 2016 as a prognostic predictor of the risks associated with colloid cysts such as obstructive hydrocephalus. CCRS is a simple 5-point clinical tool for identifying symptomatic cysts and stratifying the risk of obstructive hydrocephalus, but this tool must be externally validated before surgical indications can be established in this regard. However, the authors suggested that surgical intervention should be considered for all patients with CCRS ≥ 4, as they represent the highest-risk subgroup [23].

Surgical management of colloid cysts is still keenly debated. Currently, there are 3 available neurosurgical approaches for colloid cysts resection. These include transcortical trans/intraventricular, interhemispheric transcallosal, and transcortical endoscopic or endoscope-assisted approaches. The choice between the interhemispheric transcallosal approach and the transcortical intraventricular approach to cyst resection is mainly based on the absence or presence of hydrocephalus, respectively. Stereotactic aspiration of colloid cysts and bilateral VP shunt insertion is considered to be a second-line management option, because VP shunting historically was largely reserved for patients who were poor candidates for craniotomy, while stereotactic aspiration, unfortunately, has a high recurrence rate [22] and is limited by the variable viscosity of the colloid cyst contents, sometimes precluding aspiration. Therefore, T2WI sequences are useful for predicting the difficulty of aspiration during stereotactic or endoscopic procedures (the lower the T2WI signal, the higher the expected viscosity of the cystic contents) [24].

Colloid cysts are benign lesions with no malignant potential; therefore, surgical resection of these lesions is usually curative. However, incomplete cyst wall resection results in a remnant that can be observed over time, with only a 5%-10% symptomatic recurrence rate for cysts from wall remnants.

## Conclusion

Colloid cysts typically demonstrate hyperdense signal changes on CT scans, making them readily identifiable and easy to diagnose. However, some cysts might exhibit atypical imaging findings in the form of iso/hypodense signal changes on CT scans, making them easy to miss or misinterpret. The incidence of colloid cysts with atypical imaging features is not very high, but it is prudent to be aware of these features for early diagnosis and management to avoid morbidity and mortality associated with delayed diagnosis and treatment.

## Ethics approval and consent to participate

The approval was obtained from the Ethics Committee of Al-Azhar University Hospitals. The patient provided written informed consent to publish the case and any related data.

## Authors contribution

**M.M.** was responsible for the conception of the work, data collection, drafting the article, critical revisions, and obtaining approval of the final version of the manuscript. **D.K., A.H., and M.B.** contributed by drafting the article, and critical revisions. **M.E., A.E., A.L., A.K., M.N., and A.A.** contributed by critical revisions of the article. All authors read the final manuscript and were involved in direct patient care.

## Patient consent

The authors declare that they have obtained consent from the patient.
